# Three hours of targeted limb passive heating increases conduit artery shear rates and lowers vascular stiffness

**DOI:** 10.14814/phy2.70924

**Published:** 2026-06-12

**Authors:** Eva‐Lotte Schabbehard, Janice Habig, Justin S. Lawley

**Affiliations:** ^1^ Department of Sport Science, Division of Performance Physiology and Prevention University of Innsbruck Innsbruck Austria

**Keywords:** aortic arch, brachial artery, core temperature, limb passive heating, local thermal comfort, peripheral pulse wave velocity, shear rate, superficial femoral artery, thermal comfort

## Abstract

Cardiovascular disease remains the leading cause of mortality, primarily driven by endothelial dysfunction, which is initiated by risk factors that provoke pathological changes in endothelial function. Limb passive heating (LPH) elevates shear rate to levels comparable to mild exercise, whereby previous data has shown 38°C to be thermally comfortable. This study investigates the responses of peripheral and central arteries, as well as whole‐body and local thermal comfort during 3 h of LPH applied simultaneously to all four limbs at a skin temperature of 38°C. To achieve this, local arm and leg thermal comfort was assessed, alongside shear rate measurements in the brachial and superficial femoral arteries, as well as the ascending aortic arch at baseline and at consecutive time points throughout the 3‐h LPH exposure. Shear rate increased significantly in the brachial arteries (*p* = 0.0005) and superficial femoral arteries (*p* < 0.0001) after 3‐h LPH exposure. LPH did not significantly affect shear rate levels in the ascending aortic arch. With thermal comfort reaching 9 ± 1, the 3‐h LPH exposure was tolerable but represented the maximum limit of thermal intensity. Vascular stiffness, measured through peripheral pulse wave velocity, was significantly reduced (*p* = 0.049). This approach may serve as a suitable chronic therapeutic intervention to complement other lifestyle behaviors to improve systemic vascular stiffness and vascular function.

## 1 INTRODUCTION

Since 1945 (Office for National Statistics, 2024) CVD has been the leading cause of mortality worldwide (Statista, 2023; Tsao et al., 2023; World Health Organisation, 2022). However, an optimal treatment strategy remains elusive. CVD is largely attributed to pathological alterations in the cardiovascular system (Arnett et al., 2019; Li et al., 2022; Pizzino et al., 2017; Xu et al., 2021), with the root cause often traced to endothelial dysfunction (Baaten et al., 2023; Cyr et al., 2020; Incalza et al., 2018; Zhang, 2022), which arises from lifestyle risk factors such as smoking, alcohol consumption, physical inactivity, and an unhealthy diet. Endothelial dysfunction reduces laminar vessel wall shear stress (Cheng et al., 2019; Chistiakov et al., 2017) and diminishes nitric oxide bioavailability, which contributes to vascular stiffness (Baaten et al., 2023; Incalza et al., 2018; Jebari‐Benslaiman et al., 2022; Lubos et al., 2008; Shaito et al., 2022; Xu et al., 2021). Nitric oxide is essential for endothelial function (Incalza et al., 2018; Lubos et al., 2008) and the inhibition of atherogenic factors, specifically inflammatory cell adhesion (Bu et al., 2022; Lubos et al., 2008). Hence, the stimulation of nitric oxide production reduces the progression of endothelial dysfunction (Brinkmann et al., 2011; Cyr et al., 2020) and hypertension, both major contributors to CVD (Tsao et al., 2023), and enhances cardiovascular function (Lubos et al., 2008). In addition to peripheral conduit arteries, aortic stiffness (central stiffness) plays a major role in hypertension (Boutouyrie et al., 2021; Wilkinson et al., 2015).

Exercise increases shear rate in peripheral and central arteries (Mandell et al., 2021), and when performed regularly, improves vascular function (Black et al., 2008; Durand & Gutterman, 2014). Whole‐body passive heating (WBPH) has a positive effect on vascular function (Hoekstra et al., 2021) however, recovery time (Pelliccia et al., 2021), fitness level, limited access to appropriate facilities, and thermal intolerance in the aging population can limit the extent of shear rate exposure with exercise and WBPH. Limb passive heating (LPH) increases skin blood flow and induces downstream vasodilation. The consequence is a decrease in peripheral resistance and therefore an increase in blood velocity, shear rate, and nitric oxide (Guyton & Hall, 2006; Papaioannou & Stefanadis, 2005) production in upstream conduit arteries (Widmaier et al., 2003). Indeed, previous work has shown that acute LPH can elevate shear rate comparable to mild exercise (Amin et al., 2022) and improve vascular function (Brunt et al., 2016; Brunt & Minson, 2021; Cheng et al., 2021; Didier et al., 2022; Hoekstra et al., 2021). Thus, LPH may offer an accessible and effective alternative or complementary strategy to exercise or WBPH.

Previous studies have performed LPH intervention over 90 min with high skin temperatures ranging from 42°C to 45°C (Cheng et al., 2019, 2021; Hodges et al., 2015; Hoekstra et al., 2021; Romero et al., 2017). While these studies clearly identify that LPH can increase shear rates, the temperature range is high and generally perceived as hot and thermally uncomfortable over 45 min even when only the lower half of the leg issubmerged (Cheng et al., 2021). In contrast to these shorter more severe limb passive heating sessions, it is interesting to examine if prolonged periods (i.e., hours) of elevated shear rates may induce beneficial vascular responses, similar to those observed with low intensity exercise. Tolerance to passive heat exposure is predominantly governed by thermal perception and subjective discomfort, with cutaneous thermoreceptors and affective responses playing a primary role. In two previous studies, we identified that acute LPH of a single limb can increase conduit artery shear rate to levels similar to mild exercise (Amin et al., 2022) while maintaining local thermal comfortable (Schabbehard & Lawley, 2025). For example, targeting skin temperature at ~37°C–38°C for 10 min almost ameliorated retrograde shear rate in the brachial and superficial femoral arteries and caused a 159% and 181% increase in antegrade shear rate in the brachial and superficial femoral artery respectively, as we have shown in the paper, the findings on which this paper is based (Schabbehard & Lawley, 2025). At the same time, participants reported *feeling warm, but fairly comfortable*, and indicating that this intensity of LPH could be maintained for prolonged periods of time. A key unresolved question in the application of passive heat therapy is whether cardiovascular adaptations are primarily driven by the intensity of the thermal stimulus, the duration of exposure, or the cumulative “thermal dose” achieved. Previous studies have largely employed short‐duration, high‐temperature protocols (e.g., 42°C–45°C), which elicit robust physiological responses but may be limited by poor tolerability and reduced feasibility for repeated or long‐term use, particularly in clinical populations. In contrast, lower‐intensity heating that can be sustained for longer durations may represent a more adherence‐friendly approach, analogous to the distinction between high‐intensity and low‐intensity endurance exercise paradigms. Importantly, thermal comfort may play a central role in determining whether such interventions can be applied consistently over time, yet its importance relative to physiological stimulus remains unclear. Therefore, the present study aimed to examine whether a moderate, thermally tolerable heating stimulus (~38°C) applied over a prolonged duration (3‐h) on both arms and legs can elicit sustained haemodynamic responses, thereby providing insight into the potential balance between stimulus, duration, and tolerability in heat‐based interventions. The 3‐h duration was chosen from pilot data suggesting that with the current experimental setup, thermal comfort remained at or below “uncomfortably warm”. Thereby providing a time window to examine the intensity, duration, and tolerability of low‐level limb heating.

Physiologically, high shear rates typically cause shear‐mediated dilation of conduit arteries (Gnasso et al., 2001). Since shear rate depends on red cell velocity and artery diameter (Xiong & Zhang, 2010), prolonged heating may diminish the effectiveness of LPH by dilating the conduit arteries and reducing the absolute shear rate. Therefore, a secondary aim was to document the time course of conduit artery dilation and its impact on conduit artery shear rate. Finally, while LPH may improve peripheral vascular function, aortic stiffness (central stiffness) plays a major role in hypertension (Boutouyrie et al., 2021; Wilkinson et al., 2015). Exercise (Mandell et al., 2021) and heating paradigms employed in research studies (i.e., severe heating via hot water immersion (Didier et al., 2022) or ~50*°C* water thru a water perfused suit (Hoekstra et al., 2021)) typically cause an increase in cardiac output (Amin et al., 2021; Didier et al., 2022) and thus likely an increase in aortic shear stress. It is not clear if a modest LPH approach can cause a meaningful increase in aortic shear, which could occur through a reflex increase in heart rate and cardiac output due to the reduction in total peripheral resistance when both arms and legs are heated.

We hypothesize that whole‐body thermal comfort will increase with LPH but will be maintained at a comfortable perception over 3 h. Conduit artery blood flow and shear rate will increase with LPH, and while dilation of the conduit artery will be observed, its impact on shear rate will be minimal. Indeed, it is hypothesized that red cell velocity will continue to increase over time due to the previously observed increase in muscle temperature with chronic heating (Heinonen et al. 2011). Finally, peripheral pulse wave velocity (pPWV) will be reduced due to a decrease in peripheral vascular resistance and, whilst modest, heart rate will increase alongside an increase in aortic shear rate.

## 2 METHODS

### 2.1 Participants

Thirteen healthy participants (male = 7; female = 6) volunteered in this study. All participants were regularly engaged in physical activity (10 ± 5 *self‐reported hours of exercise per week*), nonsmokers and free of cardiac, pulmonary, or metabolic diseases. Male (age, 26 ± 1 year; height, 179.5 ± 7.9 cm; weight, 72.26 ± 9.84 kg; BMI, 22.37 ± 2.08 kg/m^2^) and female (age, 24 ± 3 years; height, 170.5 ± 5.3 cm; weight, 66.33 ± 11.9 kg; BMI, 22.94 ± 5.05 kg/m^2^) participants refrained from intense exercise, the consumption of caffeine, and alcohol for 24 h before all trials. Female participants were measured in the early follicular phase of their cycle. Informed written consent was obtained after each participant was given a verbal and written explanation of the experimental protocol and fully understood the possible risks involved in taking part in the study. The study protocols were approved by the ethics committee at University of Innsbruck and followed the principles from the Declaration of Helsinki (ethical approval number: 34/2018).

### 2.2 Experimental protocol

**FIGURE 1:**
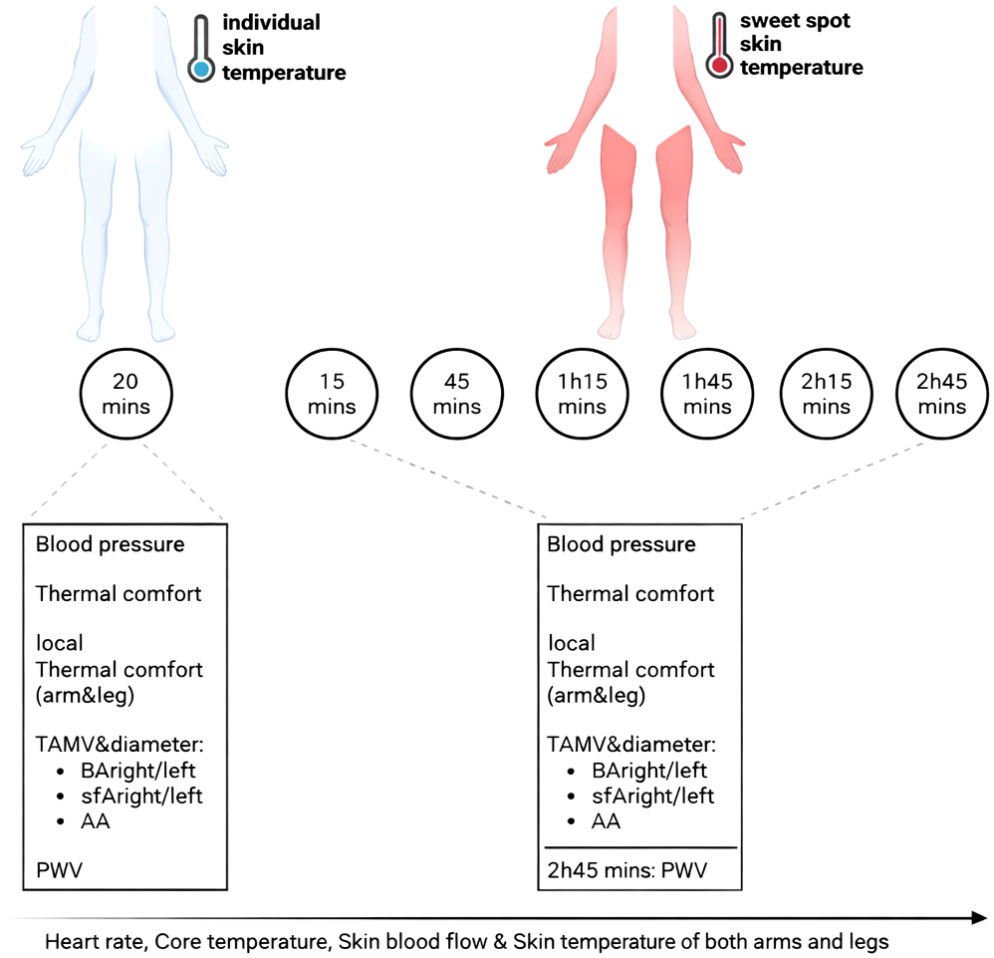
Schematic representation of the experimental protocol. Note that time corresponds to measurement time points following the initiation of the limb passive heating intervention phase.

All measurements were conducted in a quiet, environmentally controlled physiology laboratory (*ambient temperature*, 21.57°C ± 1.65°C; *humidity*, 42.42% ± 13.89%, *barometric pressure*, 944.73 ± 6.26 mmHg; time of day, 8 o'clock). After receiving ethical approval, body weight (Kern DS 150 k1; Kern & Sohn, Germany) and height were measured. Participants were asked to self‐insert a rectal probe 15 cm beyond the anal sphincter for core temperature monitoring. They then changed into shorts and sports bra for women, while men remained bare‐chested. Following this, participants were seated in a semi recumbent position (45°) on a hospital bed. PPWV was measured in accordance with the guidelines of Salvi et al. (2004) prior and post 3‐h of LPH. Briefly, the points of highest pulsation in both the carotid (in the middle of the neck) and radial arteries (close to the wrist), along with the midpoint of the suprasternal notch, were marked and verified using the tonometer probe (PulsePen tonometer®™, DiaTecne, Italy). The distances (cm) were measured using a tape measure from the marks between the carotid artery and the suprasternal notch, the carotid artery and the radial artery, and the suprasternal notch and the radial artery. After a 20‐min rest period, baseline haemodynamic data were recorded. Subsequently, all limbs were locally heated using electrical heating sleeves on the arms and water perfused tube‐lined leggings on the legs to achieve a target skin temperature of 38°C for a duration of 3 h. Measurements were repeated after 15 min, 45 min, 1 h 15 min, 1 h 45 min, 2 h 15 min and 2 h 45 min. The electrical heating sleeves were controlled using a laboratory power supply unit (PeakTech® 6225, Germany) with infinitely adjustable temperature settings, while the water perfused tube‐lined leggings were regulated by a circulating bath equipped with a digital temperature controller (PolyScience, USA) (see Figure 1).

### 2.3 Experimental measurements

#### 2.3.1 Thermal regulatory variables

Core temperature was measured with a rectal thermistor (9FR, 400 series, DeRoyal Industries Inc., USA), while forearm and leg skin temperature and skin blood flow were measured with an integrated thermistor and a laser‐Doppler flowmeter (Moor Instruments, Devon, UK). A purpose‐built probe holder (PH2) was affixed to the ventral aspect of the forearm (mid‐region) with great care to avoid contact with disruptive factors as skin hair or veins. A large‐area optic probe (LP7A/T; 2 mm ring of collecting fibers) emitting laser light (wavelength 785 nm; intensity ~1 mW) was used to measure cutaneous red blood cell flux, expressed in perfusion units. The probe was inserted into the holder until gentle contact with the skin was achieved, with care taken to avoid cutaneous compression. The probe cable was secured to the skin using a strain relief loop to ensure consistent positioning and minimize movement artifacts throughout the experimental protocol. Whole‐body, local arm and leg thermal comfort was enquired with McGinnis 13‐point scale (1 = *so cold I am helpless* to 7 = *comfortable* to 13 = *so hot I am sick and nauseate*).

#### 2.3.2 Cardiovascular variables

Heart rate was continuously recorded using three‐lead electrocardiogram (Tram‐rac, Solar 8000 M GE, Marquette, USA). Blood pressure was measured from the left arm using an electrosphygmomanometry (Tango, M2, SunTechMedical Instruments Inc., USA) with a microphone positioned over the brachial artery to detect Korotkoff sounds. PPWV was assessed using the PulsePen device (PulsePen®™, DiaTecne, Italy) whereby data quality was confirmed over 10 consecutive heart beats and visual feedback from the Pulse Wave Analysis Software (PulsePen®™, DiaTecne, Italy). PPWV was measured in accordance with the guidelines of Salvi et al. (2004) prior to and post 3‐h of LPH. Briefly, the points of highest pulsation in both the carotid and radial arteries, along with the midpoint of the suprasternal notch were marked and verified using the tonometer probe (PulsePen tonometer®TM, DiaTecne, Italy). There after the distances in mm were measured using a tape measure from the marks made between the carotid artery and the suprasternal notch, the carotid artery and the radial artery, and the suprasternal notch and the radial artery. PPWV was then quantified and calculated by obtaining high quality carotid and radial pulse waveforms over 10 consecutive heart beats using visual feedback from the Pulse Wave Analysis Software (PulsePen®™, DiaTecne, Italy). Blood pressure was measured prior and after acceptable signal acquisition.

#### 2.3.3 Peripheral ultrasonography

Brachial and superficial femoral artery time averaged mean velocity (TAMV) was measured using a 15L4A linear‐array probe (15 MHz) (uSmart 3300, Terason, United States) via continuous duplex vascular sonography (uSmart 3300, Terason, United States). Simultaneous measurements of the TAMV and arterial diameter were performed for 60 s using a custom‐made wall tracking and Doppler processing software. To ensure consistency, TAMV and diameter measurements were obtained from the same segment of the artery for each measurement, using anatomical landmarks during B‐mode imaging. TAMV was recorded at an insonation angle of 60° and was simultaneously imported, along with diameter values, into LabChart via a digital to analog converter. Antegrade and retrograde TAMV data were captured in separate channels within LabChart. All measurements were conducted by the same investigator, and basic ultrasound settings including depth, gain, power, dynamic range, and sample volume were kept constant for each participant throughout the study.

#### 2.3.4 Aortic arch ultrasonography

Aortic arch TAMV was measured using a 4V2A phased array cardiac probe (4 MHz) (uSmart 3300, Terason, United States) via continuous duplex vascular sonography (uSmart 3300, Terason, United States). Aortic arch diameters were imaged using two‐dimensional B‐mode over a period of 60 s at an insonation angle of 0°. Aortic diameter was measured in systole and diastole offline in triplicate by the same investigator. Anatomical landmarks visible during B‐mode measurements of diameter were noted to ensure consistent probe placement between baseline and all subsequent recordings. The artery section was consistently imaged just before the bifurcation of the brachiocephalic artery (ascending aorta). Basic ultrasound settings including depth, gain, power, dynamic range, and sample volume were kept constant for each participant over time.

### 2.4 Data acquisition and analysis

All continuous measurements were sampled at 250 Hz using a Powerlab (Powerlab; AD Instruments, Oxford, UK) and extracted via an offline data acquisition system (LabChart 8; AD Instruments, Oxford, UK) as averages over 60 s. Mean arterial pressure (mmHg) was calculated via the following equation: mean arterial pressure = 2/3 diastolic blood pressure + 1/3 systolic blood pressure. Aortic arch diameter was calculated with 1/3 systolic +2/3 diastolic diameter. Brachial artery, superficial femoral artery, and aortic arch blood flow (mL·min^−1^) was calculated with the following equation: blood flow = {TAMV · [π · (arterial diameter/2)2]} · 60. Antegrade or retrograde blood flow were calculated with this equation: antegrade or retrograde blood flow = {antegrade or retrograde TAMV · [π · (arterial diameter/2)2]} · 60. To calculate brachial artery, superficial femoral artery, and aortic arch shear rate (s^−1^) this equation was used: shear rate = 4 · (TAMV / diameter). The calculation of antegrade and retrograde shear rate was antegrade or retrograde shear rate = 4 · (antegrade or retrograde TAMV / diameter).

### 2.5 Statistical analyses

The Primary outcome of this study was the magnitude of effect after 3 h of limb passive heating. As such, pPWV was statistically evaluated between baseline and post 3‐h LPH using a two‐tailed paired *t*‐test. All other variables were statistically analyzed with repeated‐measurements ANOVA (Baseline and 6 heated timepoints) with the follow up Holm‐Šídák multiple comparisons tests. Initially, the primary outcome was quantified by comparing baseline and 2 h 45 min of passive heating. Thereafter, to examine the secondary question concerning the effect of time, preplanned post hoc analyses were conducted for the following intervals: baseline versus 15 min, 15 min versus 45 min, 45 min versus 1 h 15 min, 1 h 15 min versus 1 h 45 min, 1 h 45 min versus 2 h 15 min, and 2 h 15 min versus 2 h 45 min. As absolute values between the right and left arm and the right and left leg did not differ, statistical analyses were calculated for the upper and the lower extremities. For data on the differences between the right and left sides of the upper and lower extremities, see the Supporting Information. Values are expressed as mean ± standard deviation, with statistical significance set at *p* ≤ 0.05 for post hoc analysis and ANOVA *p* ≤ 0.05 for repeated‐measurement ANOVA. Prism 9 (GraphPad Software Inc., La Jolla, CA) was used for statistical analysis including the Shapiro–Wilk normality test, which all variables fulfill.

## 3 RESULTS

### 3.1 Cardiovascular effects pre and post 3 h of limb passive heating

Heart rate increased after 3‐h LPH in comparison to baseline (*p* = 0.019). Systolic blood pressure (*p* = 0.503), diastolic blood pressure (*p* = 0.897), and mean arterial pressure (*p* = 0.99) remain unchanged after 3‐h LPH. PPWV decreased after 3‐h LPH (*t*‐test, *p* = 0.049). All haemodynamic parameters in the aortic arch remained unchanged after 3‐h LPH (see Figure 4 and Tables S1–S3).

### 3.2 Core temperature and thermal comfort pre and post 3 h of limb passive heating

Core temperature (*p* = 0.04) and whole‐body thermal comfort (*p* < 0.0001) were elevated after 3‐h LPH in comparison to baseline measurements (see Tables S1–S3).

### 3.3 Local thermal comfort and arm/leg haemodynamics pre and post 3 h of limb passive heating

No meaningful differences were observed between left and right limbs for any haemodynamic variable. Therefore, data from the left and right arms and legs were averaged to improve clarity of presentation. Limb‐specific data are provided in the Supporting Information.

**FIGURE 2:**
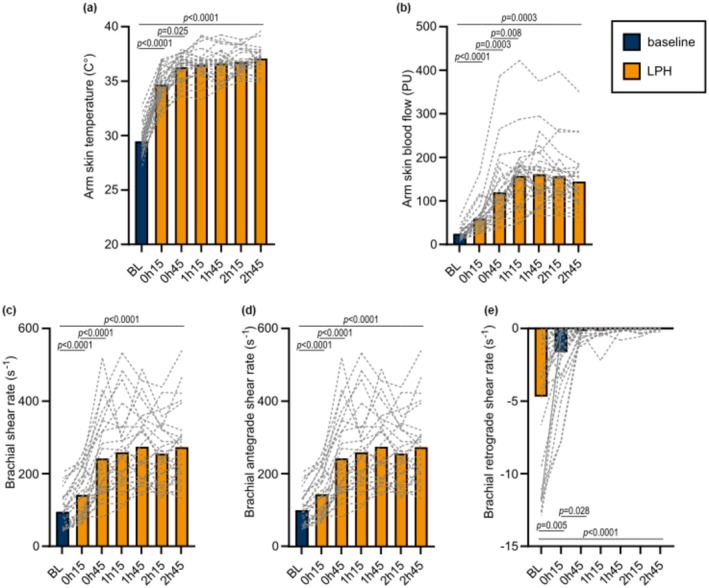
Comparison of baseline (blue) and limb passive heating (LPH) intervention (orange) across six 30‐min time intervals. Arm skin temperature (a), skin blood flow (b), Brachial arteries shear rate (c), antegrade shear rate (d), and retrograde shear rate (e). *p*‐values indicate comparisons between consecutive measurements time point and the difference between baseline and 2 h 45 min of LPH.

Local arm thermal comfort increased following 3‐h local limb passive heating (*p* < 0.0001). Arm skin temperature (*p* < 0.0001) and skin blood flow (*p* < 0.0001) were elevated postintervention. These changes were accompanied by increases in brachial artery TAMV (*p* < 0.0001), retrograde TAMV (*p* < 0.0001), and mean shear rate (*p* < 0.0001), alongside an increase in retrograde shear rate (*p* < 0.0001). Brachial artery diameter increased (*p* < 0.0001), contributing to elevations in blood flow and antegrade shear‐related indices (all *p* < 0.0001) (see Figure 2, Tables S1–S3 and Figures S1 and S2).

**FIGURE 3:**
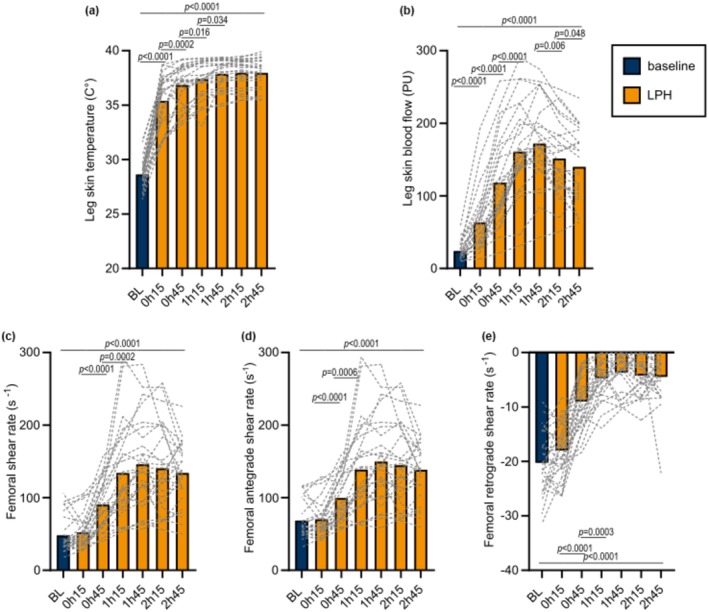
Comparison of baseline (blue) and limb passive heating (LPH) intervention (orange) across six 30‐min time intervals. Leg skin temperature (a), skin blood flow (b), superficial femoral arteries shear rate (c), antegrade shear rate (d), and retrograde shear rate (e). *p*‐values indicate comparisons between consecutive measurements time point and the difference between baseline and 2 h 45 min of LPH.

Local leg thermal comfort increased significantly following 3‐h local limb passive heating (*p* = 0.001). Skin temperature (all *p* < 0.0001) and skin blood flow (all *p* < 0.0001) of the legs were significantly elevated after 3‐h LPH. This cutaneous dilation led to an increase in TAMV (*p* < 0.0001), antegrade TAMV (*p* < 0.0001), and retrograde TAMV (*p* < 0.0001) of the superficial femoral arteries. The diameters vasodilated (*p* = 0.002 < 0.05) and blood flow (*p* < 0.0001), shear rate (*p* < 0.0001), antegrade shear rate (*p* < 0.0001), and retrograde shear rate (*p* < 0.0001) changed significantly after 3‐h limb heat exposure in both legs (see Figure 3, Tables S1–S3 and Figures S3 and S4).

### 3.4 Cardiovascular changes over time during 3 h of limb passive heating

**FIGURE 4:**
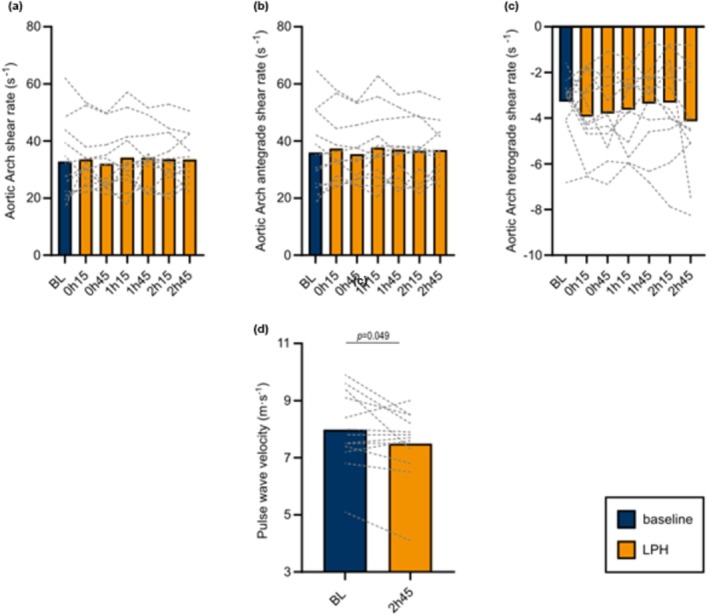
Comparison of baseline (blue) and limb passive heating (LPH) intervention (orange) across six 30‐min time intervals with repeated‐measurements ANOVA and post hoc Holm‐Šídák multiple comparisons for aortic arch shear rate (a), antegrade shear rate (b), and retrograde shear rate (c). *p*‐values indicate comparisons between baseline and 2 h 45 min of LPH. Peripheral pulse wave velocity (d) measurements during baseline and post 3‐h LPH were compared through a paired *t*‐test.

Heart rate remained relatively stable throughout the experiment, with a significant increase only observed after 2 h 45 min. Systolic, diastolic, and mean arterial pressure showed no significant changes during 3‐h LPH. Matching this, all haemodynamic parameters in the aortic arch remained unchanged throughout 3‐h LPH (see Figure 4 and Tables S1–S3).

### 3.5 Core temperature and thermal comfort changes over time during 3 h if limb passive heating

Core temperature increased throughout the heat exposure and reached steady state after 2 h 15 min (*p =* 0.219). Whole‐body thermal comfort increased between baseline and 15 min of LPH (*p* = 0.004) and between 2 h 15 min and 2 h 45 min (*p* = 0.037) (see Tables S1–S3).

### 3.6 Local thermal comfort and arm/leg haemodynamics changes over time during 3 h of limb passive heating

Local arm thermal comfort increased in the first 15 min of LPH (all *p* < 0.0001) and plateaued afterwards. Skin temperature of the arms increased between baseline and 15 min (*p* < 0.0001) and between 15 min versus 45 min (*p* = 0.025 < 0.05). Similarly, skin blood flow increased between baseline versus 15 min LPH (*p* < 0.001), between 15 min versus 45 min (*p* = 0.0003 < 0.05) and between 45 min versus 1 h 15 min (*p* = 0.008 < 0.05). Brachial arteries TAMV increased significantly between baseline and 15 min LPH (*p* < 0.0001) and between 15 min and 45 min (*p* < 0.0001) and reached a plateau afterwards. Similar patterns of change were observed in retrograde TAMV (*p* = 0.004 < 0.05; *p* = 0.04 < 0.05) (see Tables S1 and S2). The diameter increased throughout the whole 3‐h of LPH but reached the predefined statistical threshold only between baseline and 15 min LPH (*p* = 0.046 < 0.05). Blood flow (*p* < 0.0001, *p* < 0.0001, *p* = 0.005 < 0.05), shear rate (*p* < 0.0001, *p* < 0.0001) and retrograde shear rate (*p =* 0.005 < 0.05, *p* = 0.028 < 0.05) of the arms increased between baseline and 15 min LPH and between 45 min and 1 h 15 min (see Figure 2, Tables S1–S3 and Figures S1 and S2).

Local leg thermal comfort increased significantly between baseline and 15 min (*p* = 0.0003) and between 1 h 15 min and 1 h 45 min (*p* = 0.001) of LPH. Skin temperature of the legs increased after 15 min LPH (*p* < 0.0001), between 15 min and 45 min (*p* = 0.0002 < 0.05), between 45 min and 1 h 15 min (*p* = 0.016 < 0.05), and between 1 h 15 min and 1 h 45 min (*p* = 0.034 < 0.05). Skin blood flow increased after 15 min LPH (*p* < 0.0001), between 15 min and 45 min (*p* < 0.0001), between 45 min and 1 h 15 min (*p* < 0.0001), and between 2 h 15 min and 2 h 45 min (*p* = 0.048 < 0.05). Superficial femoral arteries TAMV increased significantly only after 15 min versus 45 min LPH (*p* < 0.0001) and 45 min versus 1 h 15 min (*p* = 0.0002 < 0.05), which is reflected in a similar increase in retrograde TAMV (*p* < 0.0001, *p* = 0.0003 < 0.05). The superficial femoral arteries vasodilated after 15 min LPH (*p* = 0.016 < 0.05). Together the changes in TAMV and diameter increased blood flow (*p* < 0.0001, *p* = 0.0004 < 0.05), retrograde shear rate (*p* < 0.0001, *p* = 0.0003 < 0.05), and shear rate (*p* < 0.0001, *p* = 0.0002 < 0.05) between 15 min versus 45 min and 45 min versus 1 h 15 min (see Figure 3, Tables S1–S3 and Figures S3 and S4).

## 4 DISCUSSION

The main finding of this study was that 3 h of targeted LPH caused a time‐dependent increase in conduit artery shear rate of 188% in the brachial and 180% in the femoral arteries, and an almost continuous loss of retrograde shear rate in the brachial (99.7%) and femoral (77.21%) arteries. However, whole‐body and local thermal comfort reached uncomfortable but tolerable perceptions over the course of heating. Four‐extremity LPH induced cutaneous vasodilation and an increase in blood flow through all measured conduit arteries, indicating a marked reduction in total peripheral resistance. Indeed, arterial stiffness, as estimated by pPWV, was acutely modulated after 3 h of four extremity LPH, yet blood pressure was maintained, and aortic shear rate was unaltered. The present findings should be interpreted within the broader context of a potential balance between thermal intensity, exposure duration, and tolerability. While higher‐temperature protocols may induce larger acute physiological perturbations, their practical utility may be limited if they are not well tolerated or adhered to over time. In contrast, the sustained elevations in shear rate observed in the present study suggest that moderate, tolerable heating applied over longer durations may provide a meaningful vascular stimulus while remaining feasible for repeated use. Although it remains unclear whether cumulative exposure or peak intensity is the primary driver of long‐term adaptations, our results support the concept that prolonged, tolerable heating may represent a viable alternative paradigm, particularly in populations where adherence and comfort are critical considerations.

### 4.1 Impact of 3‐h LPH on thermal comfort

The primary objective of the current study was, in contrast to previous studies, using heating protocols close to the limit of thermal tolerance, to identify if LPH could be applied for extended periods of time with beneficial changes in shear rates and vascular stiffness. Local and whole‐body thermal comfort is a major limitation when attempting to design and implement the most effective heating paradigms to improve vascular health (Schabbehard & Lawley, 2025). In this study, participants reported an increase in both whole‐body and local thermal comfort over time; however, while tolerable, it reached uncomfortable levels after a few hours, with *uncomfortably warm* (9) perceptions being reported on average. In contrast, previous research has reported that whole‐body thermal comfort increases to *hot* (10) after only ~30 min alongside an elevation in core temperatures with 42°C WBPH (Amin et al., 2021, 2022). Few studies have assessed thermal comfort during LPH. Cheng et al. (2021) performed lower limb LPH using hot water immersion (45°C) and reported that participants experienced a perception of *hot* (10) with a skin temperature exceeding 40°C. Consequently, skin temperature directly impacts the limit of local thermal comfort, which should be considered in future LPH studies. Interestingly, in the current study, a few individuals with a relatively low (27°C–28°C) resting skin temperature reported feeling “uncomfortably warm” early during the early phases of the heating paradigm, and the skin temperature had to be reduced (~35°C) to maintain thermal comfort (see Figure 2; Figures S3 and S4). Yet the delta skin temperature was similar to those individuals whose resting skin temperature was 30°C–31°C and elevated to the target temperature of 38°C. It would be interesting for future research to examine if both thermal comfort and physiological responses follow relative rather than absolute changes in skin temperature.

### 4.2 Magnitude of change in conduit haemodynamics

In our previous study (Schabbehard & Lawley, 2025), 10 min of steady state LPH caused smaller changes in brachial artery shear rate and retrograde shear rate compared to the current study (139.4 s^−1^ and ‐1.05 s versus 285.3 s^−1^ and ‐0.02 s^−1^) despite skin temperature being 2.5°C lower after 3 h LPH. In contrast, the superficial femoral arteries showed similar changes in conduit haemodynamics across both studies. The reason for this progressive rise in brachial blood flow in the arms but not the legs is not entirely clear but may represent a rise in muscle temperature with a relatively smaller muscle mass in the forearm relative to the leg (Heinonen et al., 2011; Kuipers et al., 2009). For example, Heinonen et al. (2011) increased calf skin temperature to 39.6°C with an associated increase in muscle temperature to 37.4°C, whereas Kuipers et al. (2009) increased forearm skin temperature by a similar magnitude (39.6°C), but muscle temperature increased to 38.7°C. Moreover, Rodrigues et al. (2020) measured muscle temperature with lower body passive heating using 42°C and reported that it took 115 min to recover back to baseline after immersion. While these studies are not directly comparable, it gives credence to the fact that the skeletal muscle is a major heat skin and smaller muscle mass gains heat at a quicker rate. If so, temperature‐dependent dilation of the skeletal muscle arterioles would cause an additional decrease in downstream resistance and a further increase in conduit artery blood flow beyond that caused by cutaneous vasodilation alone.

WBPH via hot water immersion or sauna substantially elevates peripheral conduit artery blood flow and thus shear rate. For example, Alali et al. (2020) performed WBPH via a water‐perfused suite with a water temperature of 48°C until core temperature increased to 1°C, and Amin et al. (2021) immersed healthy individuals in 42°C degree water for 30 min and saw a 475% increase in femoral and 272% increase in brachial shear rates, alongside a substantial rise (1°C–1.3°C) in core temperature. Moreover, Romero et al. (2017) and Cheng et al. (2021) measured femoral shear rate of around 400 s^−1^ by applying lower limb hot water immersion for 45 min. In the present study, brachial shear rates increased to 270 s^−1^ and femoral shear rates to 150 s^−1^ within ~1 h and were generally maintained for the entire duration of the protocol. While the elevation in shear rate in the current study did cause a modest vasodilation of the brachial and femoral arteries, the magnitude of change was minimal and had little impact on shear rate over time. Clearly from these data, the current approach produces much lower shear rates than what would be expected from very hot periods of WBPH or LPH, yet these shear rates can be maintained for substantially longer periods of time. Future research comparing the impact of short high intensity passive heating versus lower intensity prolonged passive heating is required, alongside perceptual measurements of thermal comfort and tolerability if these approaches are to be implemented chronically to substantially improve vascular health.

### 4.3 Aortic shear and peripheral pulse wave velocity

Central (aortic) and peripheral vascular stiffness is associated with vascular aging (Jia et al., 2019; Trache et al., 2020), systolic hypertension (Boutouyrie et al., 2021) and is a predictor of CVD (Wilkinson et al., 2015). Moreover, aortic stiffness is linked to an increase in pPWV (Torino et al., 2024). Therefore, strategies to improve central and peripheral stiffness and pPWV should improve overall cardiovascular health. Interestingly, DeBlois et al. (2020) observed an increase in microvascular function associated with high shear rates following sprint exercise, but no influence on aortic stiffness. In contrast, Gu et al. (2014) found a decrease in aortic stiffness with chronic aerobic exercise training in old rats. These data support the proposition that prolonged low‐intensity shear stimuli may have more favorable effects on certain aspects of vascular health.

Exercise causes an elevation in aortic (Cheng et al., 2003; Taylor et al., 2002) and peripheral (Amin et al., 2022) shear stress, which may explain the benefit of aerobic training on central and peripheral stiffness (Shibata et al., 2018). Moreover, WBPH typically increases cardiac output (Amin et al., 2021) and thus theoretically aortic shear stress. On the other hand, we found no increase in aortic shear with 3 h LPH at 38°C skin temperature, as heart rate remained the same during the heating phase. Interestingly, 8 weeks of WBPH therapy improves peripheral vascular function, but did not improve markers of carotid stiffness (Brunt et al., 2016). Nevertheless, this may be expected because the majority of cardiac output and elevated shear stress is directed towards the peripheral and not the cerebral arteries during passive heating. Whether WBPH therapy chronically elevates aortic shear and improves aortic stiffness has yet to be determined. Whether prolonged LPH can have a positive effect on aortic stiffness needs to be researched in long‐term intervention studies. Our findings demonstrate that 3‐h of continuous LPH of all four limbs can significantly reduce pPWV by ~6% in young, healthy individuals. Although the reduction in pPWV reached statistical significance, the magnitude of change was modest (~6%). However, this reduction is consistent with previous studies (Cheng et al., 2021; DeBlois et al., 2020; Salvi et al., 2004) investigating acute passive heating interventions. The reduction in pPWV is most plausibly explained by a decrease in cutaneous and to a modest degree, skeletal muscle and vascular resistance. While the clinical relevance of such acute changes over days, weeks and months during therapeutic applications remains to be fully established, these findings suggest that even moderate, thermally tolerable heating reduced vascular resistance and thus may influence long‐term vascular health. The reduction in total peripheral resistance was not sufficient to necessitate an increase in heart rate (and presumably cardiac output). As such, aortic shear rate was unchanged in the current intervention. Another interesting observation is that several previous studies of relatively short‐term WBPH did not observe a main effect of heating on PVW, both studies noting a correlation with baseline vascular stiffness. The current study (3 h) alongside another LPH study (45 min, Cheng et al. (2021)), observed a 6% and 8% reduction in pPWV. The role of local limb heating versus stimulus duration in reducing pPWV warrants future research. However, these data clearly highlight that LPH can be a beneficial alternative for those without access to WBPH or who have contraindications to it.

### 4.4 Limitations

In the current study, we observed small statistically significant bilateral differences in the time course to LPH. As the current study was not designed to examine bilateral differences, it is difficult to ascertain if the differences are physiologically meaningful or simply a statistical phenomenon. While this study provides valuable insights into the acute vascular effects of LPH, several limitations should be acknowledged. First, the implementation of the heating element design was relatively crude, utilizing single‐line heating elements such as heated clothing wires or water‐perfused tubes. Participants reported localized hot spots, which led to uneven heat distribution, and which may have affected thermal comfort and the magnitude of cutaneous vasodilation. Future studies should focus on optimizing heating systems to improve the balance between mean skin temperature (the resulting reduction in vascular conductance) and subjective thermal comfort. Moreover, skin temperature was measured on a skin site in between two heating elements with a single side integrated skin thermistor. While this approach negates any interference from the heating elements, the skin temperature at this site will be systematically lower than directly under the heating elements. Second, a standardized skin temperature was applied to all individuals, based on previously established mean data (Schabbehard & Lawley, 2025). This approach does not account for individual variations in thermoregulation and comfort thresholds. Indeed, as presented above, some individuals felt the same absolute skin temperature as less thermally comfortable, which appeared to be more related to the change in skin temperature. Whether shear rates and future vascular benefits follow such an individualized approach warrants further investigation. Third, this study was limited to the acute intervention, and while a significant reduction in pPWV was observed, chronic effects were not assessed. Long‐term studies are needed to evaluate the sustained impact of LPH on vascular health, including clinical endpoints such as pPWV and central pulse wave velocity, flow‐mediated dilation, and systolic blood pressure. Lastly, the study population consisted of young, healthy individuals rather than those with preexisting vascular stiffness. Although a reduction in pPWV was observed in this population, future research should investigate the effects of LPH in individuals with increased arterial stiffness to determine its potential therapeutic benefits for at‐risk populations.

## 5 CONCLUSION

Combined LPH of the arms and legs to a skin temperature of 37°C (arms) and 38°C (legs) for 3 h persistently elevated the mean shear rate in all four conduit arteries. Moreover, retrograde shear was almost abolished throughout the heating protocol. While this heating paradigm was unable to affect aortic shear rate, it lowered vascular stiffness as estimated via pPWV. These data indicate that low‐intensity LPH is an effective strategy to induce favorable acute physiological changes in peripheral conduit arteries. Moreover, these data provide the initial evidence that this approach may be a suitable chronic therapeutic intervention to complement other lifestyle behaviors to improve systemic vascular stiffness and vascular function.

## AUTHOR CONTRIBUTIONS

**Eva‐Lotte Schabbehard:** Conceptualization; data curation; formal analysis; methodology; project administration; validation. **Janice Habig:** Project administration. **Justin S. Lawley:** Conceptualization; data curation; formal analysis; funding acquisition; methodology; resources; software; supervision; validation; visualization.

## FUNDING INFORMATION

Open Access funding provided by Universitat Innsbruck.

## CONFLICT OF INTEREST STATEMENT

No conflicts of interest, financial or otherwise, are declared by the authors.

## Supporting information


**Table S1.** Data from consecutive measurement time points during baseline and limb passive heating (LPH) intervention, recorded across six intervals of 30 min. The table includes peripheral pulse wave velocity (pPWV) measurements taken at baseline and after 2 h 45 min of LPH.
**Table S2:** Post hoc Holm‐Šídák multiple comparisons derived from a repeated‐measurements ANOVA. This table presents data from consecutive measurement time points recorded during the baseline (BL) and limb passive heating (LPH) intervention, across six intervals of 30 min, as well as a comparison between baseline and 2 h 45 min of LPH. Presented *p*‐values are calculated from values shown in Table S1.
**Table S3:** Repeated‐measurements ANOVA. The analysis encompasses seven time points, ranging from baseline to consecutive measurement intervals recorded during the limb passive heating (LPH) intervention across six intervals of 30 min. Peripheral pulse wave velocity (pPWV) was specifically measured at baseline and post 3‐h of LPH, with a comparison made using a two‐tailed, paired *t*‐test. Presented *p*‐values are calculated from values shown in Table S1.
**Figure S1:** Comparison of baseline (blue) and locallimb passive heating (LPH) intervention (orange) across six 30‐min time intervals. Right arm skin temperature (A), skin blood flow (B), right brachial artery shear rate (C), antegrade shear rate (D), and retrograde shear rate (E). *p*‐values indicate comparisons between consecutive measurements time point and the difference between baseline and 2 h 45 min of LPH.
**Figure S2:** Comparison of baseline (blue) and locallimb passive heating (LPH) intervention (orange) across six 30‐min time intervals. Left arm skin temperature (A), skin blood flow (B), left brachial artery shear rate (C), antegrade shear rate (D), and retrograde PWV shear rate (E). *p*‐values indicate comparisons between consecutive measurements time point and the difference between baseline and 2 h 45 min of LPH.
**Figure S3:** Comparison of baseline (blue) and limb passive heating (LPH) intervention (orange) across six 30‐min time intervals. Right leg skin temperature (A), skin blood flow (B), right superficial femoral artery shear rate (C), antegrade shear rate (D), and retrograde shear rate (E). *p*‐values indicate comparisons between consecutive measurements time point and the difference between baseline and 2 h 45 min of LPH.
**Figure S4:** Comparison of baseline (blue) and limb passive heating (LPH) intervention (orange) across six 30‐min time intervals. Left leg skin temperature (A), skin blood flow (B), left superficial femoral artery shear rate (C), antegrade shear rate (D), and retrograde shear rate (E). *p*‐values indicate comparisons between consecutive measurements time point and the difference between baseline and 2 h 45 min of LPH.
