# Supplementary material for: Three hours of targeted limb passive heating increases conduit artery shear rates and lowers vascular stiffness
Source: Physiol Rep. 2026 Jun 12;14(11):e70924. doi: 10.14814/phy2.70924 (PMC13261082; doi:10.14814/phy2.70924)
Supplement: Supplementary file 1 — Table S1. Data from consecutive measurement time points during baseline and limb passive heating (LPH) intervention, recorded across six intervals of 30 min. The table includes peripheral pulse wave velocity (pPWV) measurements taken at baseline and after 2 h 45 min of LPH. Table S2: Post hoc Holm‐Šídák multiple comparisons derived from a repeated‐measurements ANOVA. This table presents data from consecutive measurement time points recorded during the baseline (BL) and limb passive heating (LPH) intervention, across six intervals of 30 min, as well as a comparison between baseline and 2 h 45 min of LPH. Presented p‐values are calculated from values shown in Table S1. Table S3: Repeated‐measurements ANOVA. The analysis encompasses seven time points, ranging from baseline to consecutive measurement intervals recorded during the limb passive heating (LPH) intervention across six intervals of 30 min. Peripheral pulse wave velocity (pPWV) was specifically measured at baseline and post 3‐h of LPH, with a comparison made using a two‐tailed, paired t‐test. Presented p‐values are calculated from values shown in Table S1. Figure S1: Comparison of baseline (blue) and locallimb passive heating (LPH) intervention (orange) across six 30‐min time intervals. Right arm skin temperature (A), skin blood flow (B), right brachial artery shear rate (C), antegrade shear rate (D), and retrograde shear rate (E). p‐values indicate comparisons between consecutive measurements time point and the difference between baseline and 2 h 45 min of LPH. Figure S2: Comparison of baseline (blue) and locallimb passive heating (LPH) intervention (orange) across six 30‐min time intervals. Left arm skin temperature (A), skin blood flow (B), left brachial artery shear rate (C), antegrade shear rate (D), and retrograde PWV shear rate (E). p‐values indicate comparisons between consecutive measurements time point and the difference between baseline and 2 h 45 min of LPH. Figure S3: Comparison of baseline (blue) [file PHY2-14-e70924-s001.docx]

**Three hours of targeted limb passive heating increases conduit artery shear rates and lowers vascular stiffness.**

**Eva-Lotte Schabbehard^1^, Janice Habig^1^ and Justin S. Lawley^1*^**

*Affiliations:*

^1^Department of Sport Science, Division of Performance Physiology and Prevention, University of Innsbruck, Innsbruck, Austria

**Correspondence:*

Justin S. Lawley, PhD

Department of Sport Science, Division of Performance, Physiology and Prevention, University of Innsbruck.

Fürstenweg 185, 6020

Innsbruck, Austria

Justin.Lawley@uibk.ac.at

**LIST OF ABBREVIATION**

CVD cardiovascular disease

LPH limb passive heating

pPWV peripheral pulse wave velocity

TAMV Time average mean velocity

WBPH whole-body passive heating

**SUPPLEMENTARY MATERIAL**

**TABLES**

**sTable 1.** Data from consecutive measurement time points during baseline and limb passive heating (LPH) intervention, recorded across six intervals of 30 minutes. The table includes peripheral pulse wave velocity (pPWV) measurements taken at baseline and after 2 hours 45 minutes of LPH.

|  | Baseline | | 15min | 45min | | 1h15 | | 1h45 | | 2h15 | | 2h45 |
| --- | --- | --- | --- | --- | --- | --- | --- | --- | --- | --- | --- | --- |
| pPWV (m·s^-1^) | 7.98 ± 1.32 | |  |  | |  | |  | |  | | 7.49 ± 1.23 |
| Heart rate (bpm) | 57 ± 10^†^ | | 58 ± 9 | 58 ± 9 | | 59 ± 11 | | 60 ± 8 | | 60 ± 9 | | 61 ± 8 |
| systolic blood pressure (mmHg) | 116 ± 12 | | 114 ± 16 | 113 ± 16 | | 114 ± 15 | | 116 ± 13 | | 114 ± 15 | | 113 ± 14 |
| diastolic blood pressure (mmHg) | 67 ± 6 | | 68 ± 9 | 69 ± 8 | | 66 ± 8 | | 68 ± 10 | | 70 ± 8 | | 71 ± 12 |
| Mean arterial pressure (mmHg) | 84 ± 7 | | 84 ± 11 | 84 ± 10 | | 82 ± 10 | | 84 ± 10 | | 85 ± 9 | | 85 ± 10 |
| Whole-body thermal comfort | 6 ± 1^†^* | | 7 ± 1 | 8 ± 1 | | 8 ± 1 | | 8 ± 1 | | 9 ± 1* | | 9 ± 1 |
| Core temperature (°C) | 37.09 ± 0.202^†^* | | 37.01 ± 0.185* | 36.94 ± 0.19* | | 37 ± 0.202* | | 37.11 ± 0.202* | | 37.18 ± 0.201 | | 37.21 ± 0.21 |
| Brachial artery right |  | |  |  | |  | |  | |  | |  |
| Local thermal comfort | 6 ± 1^†^* | | 8 ± 1 | 8 ± 1 | | 9 ± 1 | | 9 ± 1 | | 9 ± 1 | | 10 ± 1 |
| Skin temperature (°C) | 29.76 ± 1.21^†^* | | 34.67 ± 1.45* | 36.31 ± 1.12 | | 36.52 ± 1.43 | | 36.4 ± 1.51 | | 36.9 ± 1.2 | | 37.19 ± 1.07 |
| Skin blood flow (PU) | 23.84 ± 14.41^†^* | | 64.48 ± 38.09* | 132.3 ± 97.57 | | 161.3 ± 103.5 | | 164.7 ± 92.88 | | 166.1 ± 87.49 | | 148.3 ± 81.19 |
| TAMV (cm·s^-1^) | 7.85 ± 4.99^†^* | | 12.4 ± 5.93* | 20.07 ± 7.3 | | 22.48 ± 9.4 | | 23.35 ± 7.74 | | 21.82 ± 7.41 | | 24.07 ± 8.97 |
| Antegrade TAMV (cm·s^-1^) | 8.23 ± 4.83^†^* | | 12.54 ± 5.82* | 20.09 ± 7.28 | | 22.49 ± 9.39 | | 23.36 ± 7.74 | | 21.82 ± 7.4 | | 24.07 ± 8.97 |
| Retrograde TAMV (cm·s^-1^) | -0.39 ± 0.31^†^* | | -0.14 ± 0.2 | -0.02 ± 0.04 | | -0.01 ± 0.02 | | -0.01 ± 0.03 | | -0.01 ± 0.02 | | -0 ± 0 |
| Diameter (cm) | 0.32 ± 0.08^†^ | | 34 ± 0.07 | 0.34 ± 0.08 | | 0.35 ± 0.07 | | 0.35 ± 0.08 | | 0.36 ± 0.08 | | 0.35 ± 0.08 |
| Blood flow (ml∙min^-1^) | 44.37 ± 48.94^†^* | | 72.76 ± 55.08 | 107.8 ± 48.85* | | 131.1 ± 61 | | 137.7 ± 62.47 | | 132.7 ± 62.47 | | 146.9 ± 83.35 |
| Antegrade blood flow (ml∙min^-1^) | 46.23 ± 48.79^†^* | | 73.56 ± 54.79 | 108 ± 48.9* | | 131.2 ± 61.02 | | 137.5 ± 66.72 | | 132.7 ± 62.48 | | 146.9 ± 83.34 |
| Retrograde blood flow (ml∙min^-1^) | -1.88 ± 1.59^†^* | | -0.8 ± 1.33 | -0.18 ± 0.38 | | -0.1 ± 0.17 | | -0.08 ± 0.25 | | -0.06 ± 0.18 | | -0.01 ± 0.02 |
| Shear rate(s^-1^) | 98.87 ± 52.58^†^* | | 148.3 ± 65.67* | 255 ± 115.7 | | 267 ± 126.6 | | 284.6 ± 117 | | 259 ± 104 | | 285.3 ± 109.9 |
| Antegrade shear rate (s^-1^) | 104.1 ± 51.02^†^* | | 149.9 ± 64.37* | 255.3 ± 115.4 | | 267.2 ± 126.5 | | 284.6 ± 116.9 | | 259.1 ± 103.9 | | 285.3 ± 109.9 |
| Retrograde shear rate (s^-1^) | -5.23 ± 4.54^†^* | | -1.65 ± 2.22 | -0.24 ± 0.42 | | -0.14 ± 0.19 | | -0.1 ± 0.22 | | -0.08 ± 0.17 | | -0.02 ± 0.06 |
| Brachial artery left |  | |  |  | |  | |  | |  | |  |
| Skin temperature (°C) | 29.14 ± 1.43^†^* | | 34.64 ± 1.6* | 36.21 ± 1.25 | | 36.46 ± 1.28 | | 36.83 ± 1.17 | | 36.56 ± 0.93 | | 36.93 ± 1.15 |
| Skin blood flow (PU) | 24.69 ± 11.13^†^* | | 54.67 ± 23.58* | 106.94 ± 50.16* | | 153.30 ± 45.93 | | 157.72 ± 44.76 | | 147.15 ± 46.76 | | 140.59 ± 45.79 |
| TAMV (cm·s^-1^) | 7.46 ± 4.1^†^* | | 11.11 ± 4.68* | 18.94 ± 6.04 | | 21.17 ± 7.12 | | 22.37 ± 6.01 | | 21.59 ± 4.34 | | 22.7 ± 7.32 |
| Antegrade TAMV (cm·s^-1^) | 7.76 ± 3.86^†^* | | 11.24 ± 4.57* | 18.95 ± 6.04 | | 21.19 ± 7.13 | | 22.37 ± 6.01 | | 21.6 ± 4.34 | | 22.7 ± 7.31 |
| Retrograde TAMV (cm·s^-1^) | -0.3 ± 0.33^†^ | | -0.13 ± 0.2 | -0.01 ± 0.02 | | -0.02 ± 0.07 | | 0 ± 0 | | 0 ± 0.01 | | 0 ± 0 |
| Diameter (cm) | 0.33 ± 0.08^†^ | | 0.34 ± 0.08 | 0.35 ± 0.07 | | 0.35 ± 0.07 | | 0.35 ± 0.07 | | 0.36 ± 0.08 | | 0.36 ± 0.07 |
| Blood flow (ml∙min^-1^) | 44.2 ± 38.45^†^* | | 65.12 ± 39.51* | 108.51 ± 37.91 | | 124.94 ± 54.71 | | 135.32 ± 56.88 | | 137.93 ± 63.11 | | 144.79 ± 72.76 |
| Antegrade blood flow (ml∙min^-1^) | 45.58 ± 37.76^†^* | | 65.93 ± 39.64* | 108.6 ± 37.92 | | 125.1 ± 55.07 | | 135.3 ± 56.89 | | 138 ± 63.11 | | 144.8 ± 72.76 |
| Retrograde blood flow (ml∙min^-1^) | -1.39 ± 1.67^†^ | | -0.81 ± 1.41 | -0.06 ± 0.1 | | -0.2 ± 0.71 | | -0.02 ± 0.04 | | -0.02 ± 0.05 | | -0.01 ± 0.02 |
| Shear rate(s^-1^) | 90.84 ± 47.9^†^* | | 135.03 ± 63.32* | 229.07 ± 110.17 | | 250.82 ± 96.45 | | 263.68 ± 84.61 | | 251.52 ± 71.94 | | 260.87 ± 98.44 |
| Antegrade shear rate (s^-1^) | 94.98 ± 45.61^†^* | | 136.6 ± 62.02* | 229.2 ± 110.1 | | 251 ± 96.32 | | 263.7 ± 84.6 | | 251.6 ± 71.91 | | 260.9 ± 98.43 |
| Retrograde shear rate (s^-1^) | -4.14 ± 4.86 | | -1.58 ± 2.41 | -0.1 ± 0.2 | | -0.18 ± 0.6 | | -0.02 ± 0.05 | | -0.03 ± 0.11 | | -0.01 ± 0.03 |
| Superficial femoral artery right | |  | | |  | |  | |  | |  | |
| Local thermal comfort | 6 ± 1^†^* | | 7 ± 1 | 8 ± 1 | | 8 ± 1* | | 9 ± 1 | | 9 ± 1 | | 9 ± 1 |
| Skin temperature (°C) | 28.61 ± 1. *42^†^ | | 35.71 ± 2.44 | 36.92 ± 1.75 | | 37.27 ± 1.82 | | 37.94 ± 1.34 | | 38.05 ± 1.25 | | 38.04 ± 1.39 |
| Skin blood flow (PU) | 25.38 ± 12. *41^†^ | | 74.72 ± 49.78* | 131.39 ± 69.33* | | 174.22 ± 64.42 | | 186.08 ± 53.42* | | 166.17 ± 49.59 | | 153.45 ± 45.73 |
| TAMV (cm·s^-1^) | 6.44 ± 3^†^ | | 7.33 ± 2.85* | 12.65 ± 4.7* | | 18.62 ± 7.42 | | 19.58 ± 6.61 | | 20.67 ± 6.39 | | 19.44 ± 4.45 |
| Antegrade TAMV (cm·s^-1^) | 9.11 ± 2.9^†^ | | 9.82 ± 3.1* | 14.07 ± 4.51* | | 19.25 ± 7.14 | | 20.07 ± 6.36 | | 21.14 ± 6.08 | | 19.99 ± 4 |
| Retrograde TAMV (cm·s^-1^) | -2.67 ± 0.74^†^ | | -2.48 ± 0.72* | -1.42 ± 0.88* | | -0.63 ± 0.5 | | -0.49 ± 0.46 | | -0.47 ± 0.49 | | -0.56 ± 0.69 |
| Diameter (cm) | 0.53 ± 0.0*8 | | 0.54 ± 0.08 | 0.54 ± 0.09 | | 0.54 ± 0.09 | | 0.55 ± 0.08 | | 0.54 ± 0.09 | | 0.54 ± 0.09 |
| Blood flow (ml∙min^-1^) | 83.85 ± 37.84^†^ | | 101.39 ± 42.56* | 180.1 ± 92.37* | | 259.32 ± 127.67 | | 279.7 ± 122.05 | | 289.37 ± 115.25 | | 282.17 ± 128.69 |
| Antegrade blood flow (ml∙min^-1^) | 120.7 ± 46.83^†^ | | 138 ± 55.56* | 202 ± 99.37* | | 268.6 ± 127.1 | | 287 ± 120.8 | | 297.3 ± 117.1 | | 289.6 ± 127.3 |
| Retrograde blood flow (ml∙min^-1^) | -36.82 ± 19.44^†^ | | -36.59 ± 18.69* | -21.95 ± 18.29* | | -9.25 ± 8.9 | | -7.32 ± 7.45 | | -7.9 ± 9 | | -7.43 ± 7.83 |
| Shear rate(s^-1^) | 50.21 ± 25.27^†^ | | 55.62 ± 23.97* | 95.47 ± 38.79* | | 142 ± 63.7 | | 146.86 ± 56.3 | | 156.99 ± 58.29 | | 145.56 ± 34.82 |
| Antegrade shear rate (s^-1^) | 70.61 ± 24.77^†^ | | 73.95 ± 25.96* | 105.7 ± 36.55* | | 146.6 ± 61.7 | | 150.4 ± 54.75 | | 160.2 ± 55.81 | | 149.9 ± 32.48 |
| Retrograde shear rate (s^-1^) | -20.37 ± 5.43^†^ | | -18.29 ± 4.74* | -10.24 ± 5.55* | | -4.63 ± 3.42 | | -3.55 ± 3.25 | | -3.21 ± 3.19 | | -4.36 ± 5.97 |
| Superficial femoral artery left | | | | | | | | | | | | |
| Skin temperature (°C) | 28.63 ± 1.33^†^* | | 35.04 ± 2.53* | 36.76 ± 2* | | 37.48 ± 1.55* | | 37.79 ± 1.38 | | 37.85 ± 1.38 | | 37.85 ± 1.37 |
| Skin blood flow (PU) | 22.05 ± 10.77^†^* | | 51.52 ± 26.93* | 104.72 ± 48.42* | | 147.57 ± 56.49* | | 157.47 ± 57.7* | | 136.42 ± 51.75* | | 126.44 ± 46.94 |
| TAMV (cm·s^-1^) | 5.97 ± 3.11^†^ | | 6.57 ± 2.41* | 11.56 ± 3.51 | | 16.43 ± 7.83 | | 18.99 ± 8.16 | | 16.1 ± 5.79 | | 16.34 ± 4.32 |
| Antegrade TAMV (cm·s^-1^) | 8.65 ± 3.08^†^ | | 8.97 ± 2.63* | 12.63 ± 3.48 | | 17.1 ± 7.54 | | 19.53 ± 7.8 | | 16.85 ± 5.42 | | 16.98 ± 4.14 |
| Retrograde TAMV (cm·s^-1^) | -2.68 ± 0.88^†^ | | -2.4 ± 0.65* | -1.07 ± 0.56 | | -0.67 ± 0.55 | | -0.54 ± 0.58 | | -0.75 ± 0.53 | | -0.64 ± 0.4 |
| Diameter (cm) | 0.54 ± 0.08 | | 0.55 ± 0.08 | 0.55 ± 0.09 | | 0.54 ± 0.09 | | 0.54 ± 0.08 | | 0.55 ± 0.09 | | 0.56 ± 0.1 |
| Blood flow (ml∙min^-1^) | 80.81 ± 40.05^†^ | | 95.22 ± 41.71* | 172.52 ± 84.54 | | 224.4 ± 106.07 | | 262.49 ± 133.09 | | 222.16 ± 76.22 | | 239.6 ± 76.15 |
| Antegrade blood flow (ml∙min^-1^) | 119.6 ± 49.66^†^ | | 130.3 ± 49.19* | 189.1 ± 88.29 | | 234.8 ± 103.9 | | 270.8 ± 130.5 | | 234.3 ± 76.69 | | 249.9 ± 76.68 |
| Retrograde blood flow (ml∙min^-1^) | -38.76 ± 22.27^†^ | | -35.02 ± 13.78* | -16.49 ± 11.22 | | -10.38 ± 9.27 | | -8.33 ± 9.84 | | -12.07 ± 10.53 | | -10.26 ± 8.09 |
| Shear rate(s^-1^) | 45.71 ± 25.37^†^ | | 48.81 ± 20.51* | 85.3 ± 27.14* | | 125.8 ± 65.96 | | 144.69 ± 68.43 | | 123.58 ± 57.35 | | 122.22 ± 47.03 |
| Antegrade shear rate (s^-1^) | 65.78 ± 25.49^†^ | | 66.52 ± 23.46* | 93.06 ± 27.15 | | 130.6 ± 64.07 | | 148.6 ± 66.07 | | 128.8 ± 54.93 | | 126.8 ± 46.6 |
| Retrograde shear rate (s^-1^) | -20.05 ± 6^†^ | | 17.7 ± 5.32* | -7.74 ± 4 | | -4.81 ± 3.95 | | -3.86 ± 4.08 | | -5.25 ± 3.49 | | -4.57 ± 2.81 |
| Aortic Arch |  | |  |  | |  | |  | |  | |  |
| TAMV (cm·s^-1^) | 15.19 ± 5.62 | | 15.41 ± 4.03 | 14.88 ± 4.42 | | 15.73 ± 4.92 | | 15.63 ± 4.31 | | 15.74 ± 4.22 | | 15.48 ± 3.91 |
| Antegrade TAMV (cm·s^-1^) | 16.65 ± 5.78 | | 17.09 ± 4.42 | 16.42 ± 4.61 | | 17.32 ± 5.16 | | 16.89 ± 4.59 | | 17.05 ± 4.61 | | 16.99 ± 3.98 |
| Retrograde TAMV (cm·s^-1^) | -1.39 ± 0.65 | | -1.78 ± 0.62 | -1.74 ± 0.73 | | -1.65 ± 0.68 | | -1.47 ± 0.72 | | -1.47 ± 0.9 | | -1.87 ± 0.96 |
| Diameter (cm) | 1.89 ± 0.24 | | 1.86 ± 0.2 | 1.87 ± 0.15 | | 1.86 ± 0.18 | | 1.84 ± 0.19 | | 1.89 ± 0.19 | | 1.85 ± 0.16 |
| Blood flow (ml∙min^-1^) | 2585 ± 1197 | | 2535 ± 852.9 | 2468 ± 871.4 | | 2589 ± 1018 | | 2540 ± 1018 | | 2683 ± 1005 | | 2546 ± 897.8 |
| Antegrade blood flow (ml∙min^-1^) | 2835 ± 1268 | | 2799 ± 941.1 | 2717 ± 894.1 | | 2841 ± 1042 | | 2736 ± 999 | | 2889 ± 1034 | | 2787 ± 921.2 |
| Retrograde blood flow (ml∙min^-1^) | -256.8 ± 98.95 | | -284.8 ± 102.4 | -282.8 ± 111.5 | | -263.9 ± 106.7 | | -230.3 ± 82.58 | | -238.9 ± 108.8 | | -294.8 ± 147.3 |
| Shear rate(s^-1^) | 32.76 ± 13.64 | | 33.57 ± 10.15 | 32.02 ± 9.85 | | 34.18 ± 11.61 | | 34.15 ± 9.65 | | 33.6 ± 9.54 | | 33.5 ± 8.31 |
| Antegrade shear rate (s^-1^) | 35.93 ± 14.21 | | 37.35 ± 11.36 | 35.38 ± 10.53 | | 37.69 ± 12.54 | | 36.99 ± 10.62 | | 36.53 ± 10.89 | | 35.83 ± 8.68 |
| Retrograde shear rate (s^-1^) | -3.28 ± 1.31 | | -3.93 ± 1.63 | -3.79 ± 1.74 | | -3.63 ± 1.67 | | -3.37 ± 1.8 | | -3.32 ± 2.12 | | -4.13 ± 2.26 |

Data are presented as mean ± standard deviation, TAMV, time average mean velocity. P-values of post-hoc Holm--Šídák multiple comparisons (Table 2) and repeated-measured ANOVA (Table 3) are presented below. *, p-values < 0.05 for following comparisons: BL versus 15min, 15min versus 45min, 45min versus 1h15, 1h15 versus 1h45, 1h45 versus 2h15, 2h15 versus 2h45; †, p-values < 0.05 for following comparison: BL versus 2h45.

**sTable 2.** Post-hoc Holm-Šídák multiple comparisons derived from a repeated-measurements ANOVA. This table presents data from consecutive measurement time points recorded during the baseline (BL) and limb passive heating (LPH) intervention, across six intervals of 30 minutes, as well as a comparison between baseline and 2 hours 45 minutes of LPH. Presented p-values are calculated from values shown in table 1.

|  | BL vs 15min | 15min vs 45min | | 45min vs 1h15 | 1h15 vs 1h45 | 1h45 vs 2h15 | 2h15 vs 2h45 | BL vs 2h45 |
| --- | --- | --- | --- | --- | --- | --- | --- | --- |
| Heart rate (bpm) | 0.953 | | 0.953 | 0.953 | 0.953 | 0.953 | 0.413 | 0.019 |
| systolic blood pressure (mmHg) | 0.62 | | 0.929 | 0.929 | 0.503 | 0.503 | 0.894 | 0.503 |
| diastolic blood pressure (mmHg) | 0.897 | | 0.897 | 0.422 | 0.897 | 0.897 | 0.897 | 0.897 |
| Mean arterial pressure (mmHg) | >0.999 | | 0.99 | 0.63 | 0.63 | 0.99 | 0.996 | 0.99 |
| Whole-body thermal comfort | 0.004 | | 0.175 | 0.469 | 0.561 | 0.561 | 0.037 | <0.0001 |
| Core temperature (°C) | 0.034 | | 0.03 | 0.04 | 0.0004 | 0.011 | 0.219 | 0.04 |
| Brachial artery right |  | |  |  |  |  |  |  |
| Local thermal comfort | <0.0001 | | 0.419 | 0.214 | 0.419 | 0.636 | 0.214 | <0.0001 |
| Skin temperature (°C) | <0.0001 | | 0.012 | 0.747 | 0.747 | 0.221 | 0.691 | <0.0001 |
| Skin blood flow (PU) | 0.001 | | 0.024 | 0.225 | 0.982 | 0.982 | 0.111 | 0.0003 |
| TAMV (cm·s^-1^) | 0.0002 | | 0.009 | 0.144 | 0.522 | 0.346 | 0.182 | <0.0001 |
| Antegrade TAMV (cm·s^-1^) | 0.0004 | | 0.009 | 0.146 | 0.523 | 0.345 | 0.181 | 0.0001 |
| Retrograde TAMV (cm·s^-1^) | 0.012 | | 0.259 | 0.705 | 0.705 | 0.705 | 0.705 | 0.005 |
| Diameter (cm) | 0.202 | | 0.846 | 0.233 | 0.774 | 0.26 | 0.494 | 0.018 |
| Blood flow (ml∙min^-1^) | 0.001 | | 0.061 | 0.028 | 0.636 | 0.636 | 0.553 | 0.0003 |
| Antegrade blood flow (ml∙min^-1^) | 0.002 | | 0.064 | 0.028 | 0.634 | 0.634 | 0.553 | 0.0004 |
| Retrograde blood flow (ml∙min^-1^) | 0.009 | | 0.466 | 0.745 | 0.849 | 0.745 | 0.745 | 0.008 |
| Shear rate(s^-1^) | 0.0003 | | 0.009 | 0.683 | 0.683 | 0.398 | 0.078 | 0.0002 |
| Antegrade shear rate (s^-1^) | 0.001 | | 0.009 | 0.684 | 0.684 | 0.398 | 0.077 | 0.0002 |
| Retrograde shear rate (s^-1^) | 0.028 | | 0.169 | 0.662 | 0.723 | 0.723 | 0.662 | 0.009 |
| Brachial artery left |  | |  |  |  |  |  |  |
| Skin temperature (°C) | <0.0001 | | 0.002 | 0.375 | 0.37 | 0.375 | 0.17 | <0.0001 |
| Skin blood flow (PU) | 0.001 | | 0.011 | 0.015 | 0.752 | 0.752 | 0.752 | <0.0001 |
| TAMV (cm·s^-1^) | 0.001 | | 0.001 | 0.628 | 0.697 | 0.721 | 0.721 | <0.0001 |
| Antegrade TAMV (cm·s^-1^) | 0.002 | | 0.001 | 0.625 | 0.711 | 0.722 | 0.722 | <0.0001 |
| Retrograde TAMV (cm·s^-1^) | 0.226 | | 0.226 | 0.854 | 0.808 | 0.854 | 0.854 | 0.048 |
| Diameter (cm) | 0.247 | | 0.239 | 0.867 | 0.51 | 0.632 | 0.867 | 0.001 |
| Blood flow (ml∙min^-1^) | 0.016 | | <0.0001 | 0.189 | 0.516 | 0.842 | 0.802 | <0.0001 |
| Antegrade blood flow (ml∙min^-1^) | 0.013 | | <0.0001 | 0.188 | 0.541 | 0.841 | 0.803 | <0.0001 |
| Retrograde blood flow (ml∙min^-1^) | 0.819 | | 0.344 | 0.819 | 0.819 | 0.859 | 0.819 | 0.077 |
| Shear rate(s^-1^) | 0.003 | | 0.003 | 0.85 | 0.85 | 0.85 | 0.85 | 0.0001 |
| Antegrade shear rate (s^-1^) | 0.003 | | 0.003 | 0.848 | 0.848 | 0.848 | 0.848 | 0.0002 |
| Retrograde shear rate (s^-1^) | 0.196 | | 0.196 | 0.86 | 0.805 | 0.86 | 0.86 | 0.067 |
| Superficial femoral artery right | | | | | | | | |
| Local thermal comfort | 0.0003 | | 0.19 | 0.95 | 0.001 | 0.95 | 0.149 | 0.001 |
| Skin temperature (°C) | <0.0001 | | 0.063 | 0.516 | 0.241 | 0.516 | 0.864 | <0.0001 |
| Skin blood flow (PU) | 0.004 | | 0.001 | 0.002 | 0.271 | 0.018 | 0.202 | <0.0001 |
| TAMV (cm·s^-1^) | 0.41 | | 0.001 | 0.003 | 0.483 | 0.41 | 0.506 | <0.0001 |
| Antegrade TAMV (cm·s^-1^) | 0.414 | | 0.004 | 0.009 | 0.56 | 0.368 | 0.56 | <0.0001 |
| Retrograde TAMV (cm·s^-1^) | 0.712 | | 0.002 | 0.002 | 0.576 | 0.914 | 0.914 | <0.0001 |
| Diameter (cm) | 0.021 | | 0.963 | 0.893 | 0.211 | 0.893 | 0.963 | 0.286 |
| Blood flow (ml∙min^-1^) | 0.092 | | 0.005 | 0.002 | 0.317 | 0.572 | 0.79 | 0.001 |
| Antegrade blood flow (ml∙min^-1^) | 0.064 | | 0.015 | 0.004 | 0.357 | 0.489 | 0.763 | 0.001 |
| Retrograde blood flow (ml∙min^-1^) | 0.98 | | 0.002 | 0.012 | 0.606 | 0.976 | 0.98 | 0.001 |
| Shear rate(s^-1^) | 0.568 | | 0.001 | 0.008 | 0.665 | 0.272 | 0.665 | <0.0001 |
| Antegrade shear rate (s^-1^) | 0.791 | | 0.004 | 0.018 | 0.791 | 0.253 | 0.791 | <0.0001 |
| Retrograde shear rate (s^-1^) | 0.579 | | 0.003 | 0.001 | 0.579 | 0.805 | 0.805 | <0.0001 |
| Superficial femoral artery left |  | |  |  |  |  |  |  |
| Skin temperature (°C) | <0.0001 | | 0.004 | 0.004 | 0.005 | 0.639 | 0.93 | <0.0001 |
| Skin blood flow (PU) | 0.001 | | 0.001 | 0.0002 | 0.029 | 0.025 | 0.029 | <0.0001 |
| TAMV (cm·s^-1^) | 0.621 | | 0.0004 | 0.059 | 0.059 | 0.122 | 0.791 | <0.0001 |
| Antegrade TAMV (cm·s^-1^) | 0.862 | | 0.003 | 0.075 | 0.075 | 0.139 | 0.868 | 0.0001 |
| Retrograde TAMV (cm·s^-1^) | 0.516 | | 0.0002 | 0.125 | 0.516 | 0.322 | 0.516 | <0.0001 |
| Diameter (cm) | 0.24 | | 0.932 | 0.405 | 0.932 | 0.672 | 0.133 | 0.087 |
| Blood flow (ml∙min^-1^) | 0.375 | | 0.005 | 0.152 | 0.156 | 0.267 | 0.375 | <0.0001 |
| Antegrade blood flow (ml∙min^-1^) | 0.457 | | 0.016 | 0.228 | 0.228 | 0.309 | 0.457 | 0.0001 |
| Retrograde blood flow (ml∙min^-1^) | 0.508 | | 0.002 | 0.11 | 0.48 | 0.3 | 0.508 | 0.001 |
| Shear rate(s^-1^) | 0.787 | | 0.001 | 0.043 | 0.07 | 0.102 | 0.852 | 0.001 |
| Antegrade shear rate (s^-1^) | 0.939 | | 0.004 | 0.052 | 0.088 | 0.115 | 0.939 | 0.002 |
| Retrograde shear rate (s^-1^) | 0.374 | | 0.0002 | 0.16 | 0.453 | 0.369 | 0.519 | <0.0001 |
| Aortic Arch |  | |  |  |  |  |  |  |
| TAMV (cm·s^-1^) | 0.999 | | 0.908 | 0.739 | 0.999 | 0.999 | 0.991 | 0.999 |
| Antegrade TAMV (cm·s^-1^) | 0.995 | | 0.988 | 0.53 | 0.988 | 0.995 | 0.995 | 0.995 |
| Retrograde TAMV (cm·s^-1^) | 0.395 | | 0.903 | 0.903 | 0.583 | 0.992 | 0.474 | 0.583 |
| Diameter (cm) | 0.808 | | 0.921 | 0.921 | 0.826 | 0.599 | 0.819 | 0.819 |
| Blood flow (ml∙min^-1^) | 0.991 | | 0.991 | 0.953 | 0.991 | 0.945 | 0.717 | 0.991 |
| Antegrade blood flow (ml∙min^-1^) | 0.946 | | 0.991 | 0.946 | 0.946 | 0.946 | 0.946 | 0.966 |
| Retrograde blood flow (ml∙min^-1^) | 0.972 | | 0.972 | 0.972 | 0.671 | 0.972 | 0.62 | 0.972 |
| Shear rate(s^-1^) | 0.995 | | 0.677 | 0.57 | 0.997 | 0.995 | 0.997 | 0.995 |
| Antegrade shear rate (s^-1^) | 0.995 | | 0.612 | 0.389 | 0.995 | 0.995 | 0.995 | 0.995 |
| Retrograde shear rate (s^-1^) | 0.773 | | 0.953 | 0.953 | 0.82 | 0.953 | 0.657 | 0.791 |

Significance level, *p* < 0.05. TAMV, time average mean velocity.

**sTable 3.** Repeated-measurements ANOVA. The analysis encompasses seven time points, ranging from baseline to consecutive measurement intervals recorded during the limb passive heating (LPH) intervention across six intervals of 30 minutes. Peripheral pulse wave velocity (pPWV) was specifically measured at baseline and post 3-hours of LPH, with a comparison made using a two-tailed, paired t-test. Presented p-values are calculated from values shown in table 1.

|  | Total | Brachial artery right | Brachial artery left | Femoral artery right | Femoral artery left | Aortic Arch |
| --- | --- | --- | --- | --- | --- | --- |
| pPWV (m·s^-1^) | 0.049 |  |  |  |  |  |
| Hear rate (bpm) | 0.018 |  |  |  |  |  |
| systolic blood pressure (mmHg) | 0.328 |  |  |  |  |  |
| diastolic blood pressure (mmHg) | 0.368 |  |  |  |  |  |
| Mean arterial pressure (mmHg) | 0.572 |  |  |  |  |  |
| Whole-body thermal comfort | <0.0001 |  |  |  |  |  |
| Core temperature (°C) | <0.0001 |  |  |  |  |  |
| Local arm thermal comfort | <0.0001 |  |  |  |  |  |
| Local leg thermal comfort | <0.0001 |  |  |  |  |  |
| Skin temperature (°C) |  | <0.0001 | <0.0001 | <0.0001 | <0.0001 |  |
| Skin blood flow (PU) |  | <0.0001 | <0.0001 | <0.0001 | <0.0001 |  |
| TAMV (cm·s^-1^) |  | <0.0001 | <0.0001 | <0.0001 | <0.0001 | 0.786 |
| Antegrade TAMV (cm·s^-1^) |  | <0.0001 | <0.0001 | <0.0001 | <0.0001 | 0.721 |
| Retrograde TAMV (cm·s^-1^) |  | 0.001 | 0.005 | <0.0001 | <0.0001 | 0.197 |
| Diameter (cm) |  | 0.001 | <0.0001 | 0.052 | 0.018 | 0.532 |
| Blood flow (ml∙min^-1^) |  | <0.0001 | <0.0001 | <0.0001 | <0.0001 | 0.804 |
| Antegrade blood flow (ml∙min^-1^) |  | <0.0001 | <0.0001 | <0.0001 | <0.0001 | 0.751 |
| Retrograde blood flow (ml∙min^-1^) |  | 0.002 | 0.011 | <0.0001 | <0.0001 | 0.415 |
| Shear rate(s^-1^) |  | <0.0001 | <0.0001 | <0.0001 | <0.0001 | 0.714 |
| Antegrade shear rate (s^-1^) |  | <0.0001 | <0.0001 | <0.0001 | <0.0001 | 0.639 |
| Retrograde shear rate (s^-1^) |  | 0.001 | 0.008 | <0.0001 | <0.0001 | 0.37 |

Significance level, *P* < 0.05, TAMV, time average mean velocit


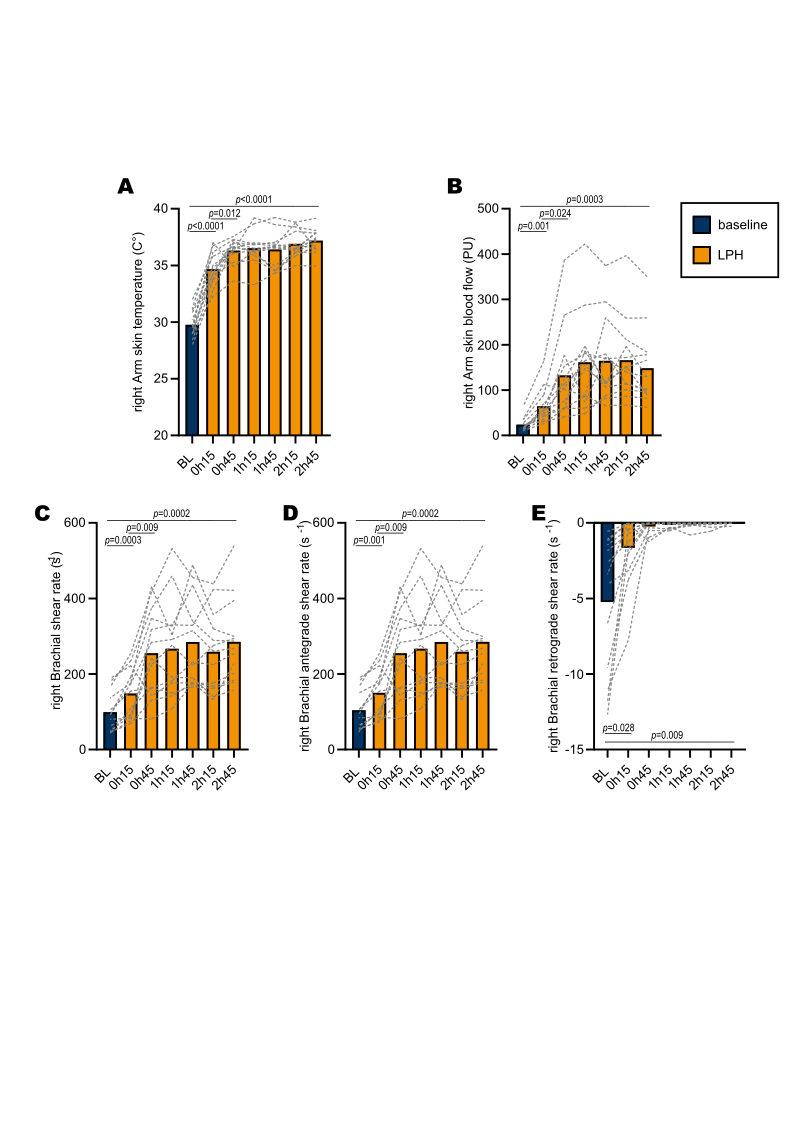


**sFigure 1.** Comparison of baseline (blue) and locallimb passive heating (LPH) intervention (orange) across six 30-minute time intervals. Right arm skin temperature (A), skin blood flow (B), right brachial artery shear rate (C), antegrade shear rate (D), and retrograde shear rate (E). *P*-values indicate comparisons between consecutive measurements time point and the difference between baseline and 2 hours 45 minutes of LPH.


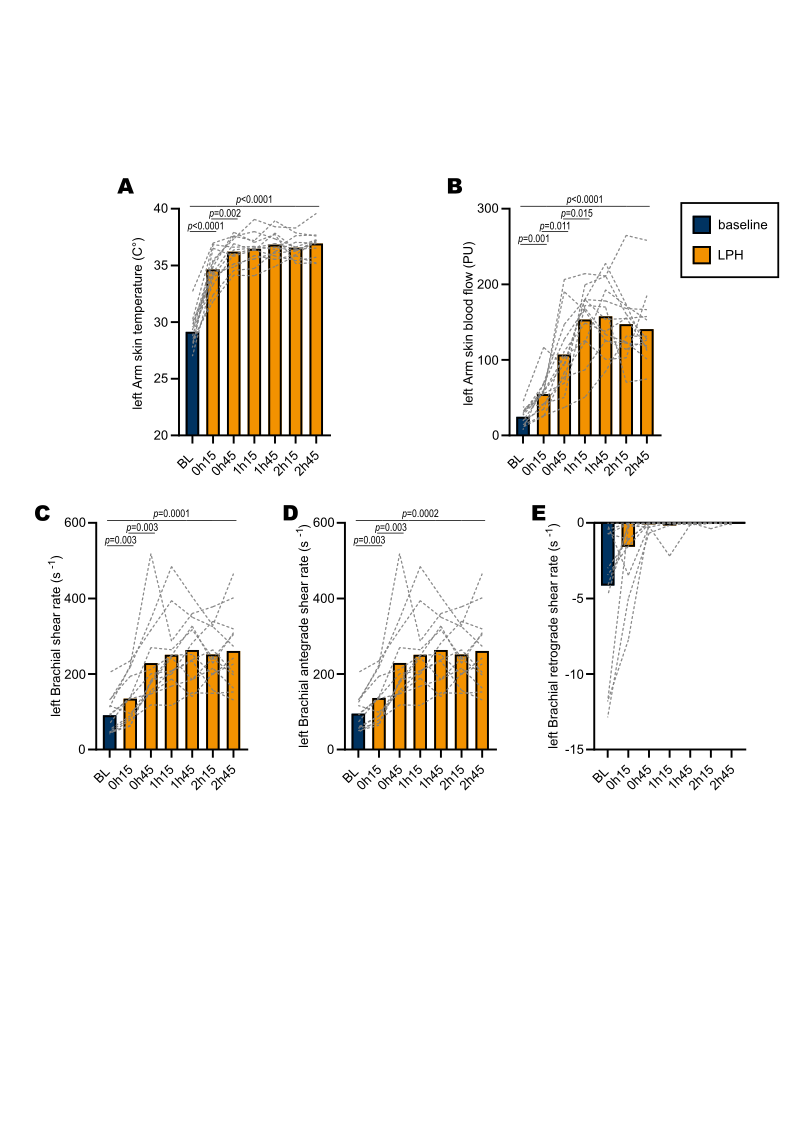


**sFigure 2.** Comparison of baseline (blue) and locallimb passive heating (LPH) intervention (orange) across six 30-minute time intervals. Left arm skin temperature (A), skin blood flow (B), left brachial artery shear rate (C), antegrade shear rate (D), and retrograde PWV shear rate (E). *P*-values indicate comparisons between consecutive measurements time point and the difference between baseline and 2 hours 45 minutes of LPH.


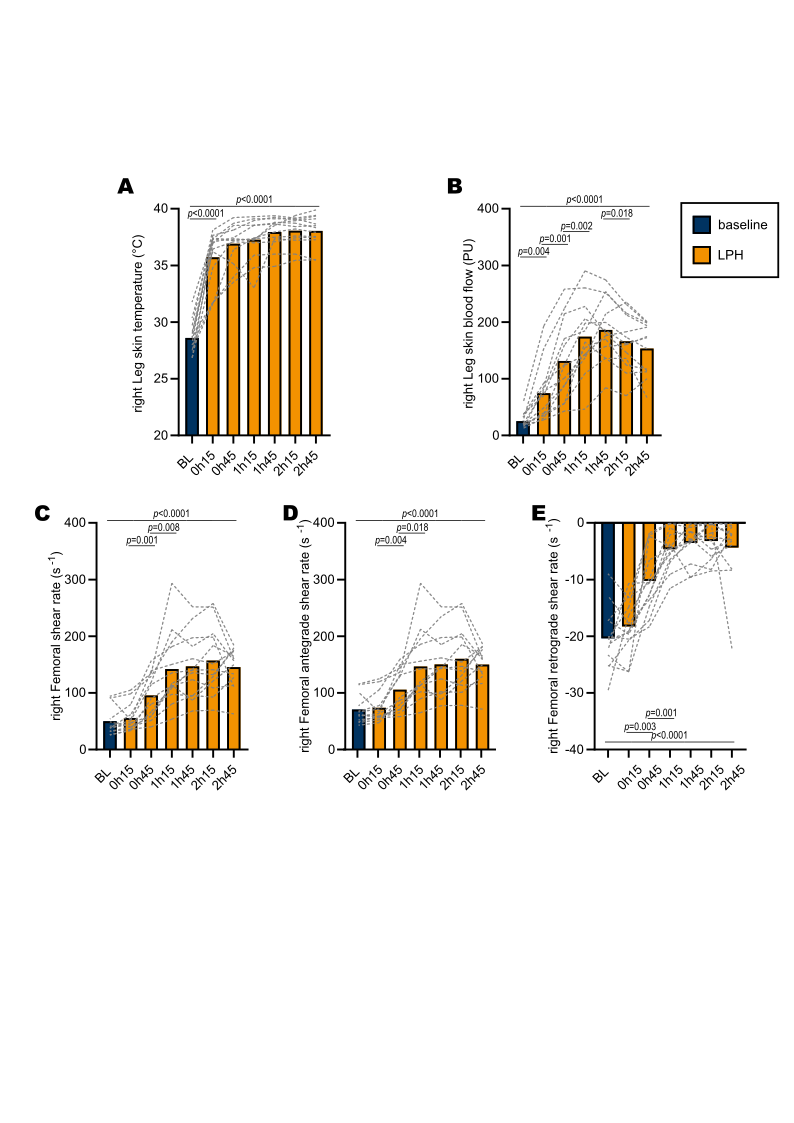


**sFigure 3.** Comparison of baseline (blue) and limb passive heating (LPH) intervention (orange) across six 30-minute time intervals. Right leg skin temperature (A), skin blood flow (B), right superficial femoral artery shear rate (C), antegrade shear rate (D), and retrograde shear rate (E). *P*-values indicate comparisons between consecutive measurements time point and the difference between baseline and 2 hours 45 minutes of LPH.


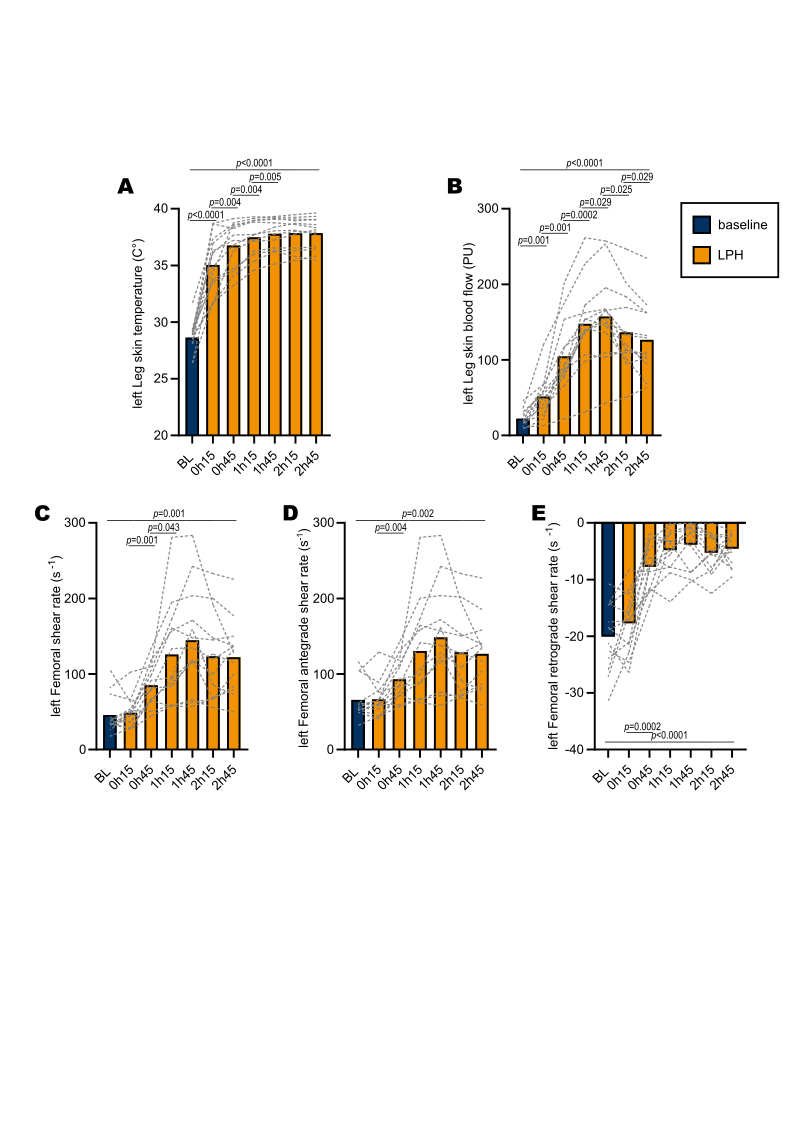


**sFigure 4.** Comparison of baseline (blue) and limb passive heating (LPH) intervention (orange) across six 30-minute time intervals. Left leg skin temperature (A), skin blood flow (B), left superficial femoral artery shear rate (C), antegrade shear rate (D), and retrograde shear rate (E). P-values indicate comparisons between consecutive measurements time point and the difference between baseline and 2 hours 45 minutes of LPH.
